# The relationship between pregnant women's perception of traumatic birth and their mode of delivery preference

**DOI:** 10.1590/1806-9282.20241835

**Published:** 2025-07-07

**Authors:** Esra Ünal, Şükrü Can Karaman, Sevim Yildirim, Hatice Aslıhan Hacimuhittinoğullari

**Affiliations:** 1 Osmaniye Korkut Ata University, Faculty of Health Sciences, Nursing Department – Osmaniye, Türkiye.

**Keywords:** Pregnancy, Psychological trauma, Perception, Natural childbirth, Attitudes

## Abstract

**OBJECTIVE:**

This study aims to examine the relationship between pregnant women's perception of traumatic birth and their preference for the mode of delivery.

**METHODS:**

This cross-sectional study was conducted between April 12, 2023, and June 22, 2023, in the obstetrics and gynecology clinic of a state hospital in Turkey. The study population consisted of pregnant women who applied to the clinic during the study period. The inclusion criteria were being 18 years or older, having a singleton pregnancy between the 20th and 40th gestational weeks, and having no high-risk pregnancy conditions. The exclusion criteria included pregnancy through assisted reproductive techniques and the presence of intellectual disabilities. Participants meeting the inclusion criteria were selected through a non-probability random sampling method. Data were collected using a sociodemographic questionnaire, the traumatic childbirth perception scale, and the belief scale for normal delivery.

**RESULTS:**

Among the participants, 71.8% preferred cesarean section over vaginal delivery. A significant negative correlation was found between the perception of traumatic birth and the belief in and preference for vaginal delivery (r=-0.427, p<0.001). Pregnant women with a higher perception of traumatic birth were more likely to choose cesarean delivery.

**CONCLUSION:**

The findings suggest that a higher perception of traumatic birth is associated with a decreased preference for vaginal delivery. Addressing these concerns may help support informed decision-making regarding childbirth.

## INTRODUCTION

The concept of traumatic birth perception refers to a woman's subjective understanding of childbirth as a potentially harmful experience for herself or her infant, encompassing all stages of the reproductive process^1^. The perception of birth can be shaped by a range of factors, including but not limited to traumatic birth encounters, individual personality traits, emotional states, societal perspectives on childbirth, educational attainment, and cultural norms and values^1,2^. Academic research reveals that those who prefer vaginal birth put forward more than one reason. These include less postpartum discomfort and bleeding, vaginal birth being seen as a more natural and desirable option for the baby, an easier return to normal daily activities, and earlier discharge from the hospital. Several factors contribute to the preference for cesarean delivery. These include medical indications and the decision made by the attending physician, the potential for reduced pain and increased comfort for the mother, the prioritization of the baby's safety, the desire to avoid perineal tear, the potential for a shorter operation time, and the ease of care during the procedure^3^.

A community-based study has investigated the correlation between the dread of labor and the choice of elective cesarean, uncovering a positive relationship between the fear of childbirth and the inclination toward cesarean birth. Furthermore, a study conducted by Størksen et al.^4^ revealed that individuals with previous unpleasant birth experiences were more likely to choose for a cesarean birth. According to a systematic review conducted by Jenabi et al.^5^, various factors were identified as reasons for maternal requests for elective cesareans. These factors include the fear of experiencing pain during childbirth, concerns about potential injury or death of the infant, pelvic floor and vaginal trauma, recommendations from healthcare professionals, and previous negative birth experiences. Based on this research, it can be asserted that the preference for delivery style is influenced by individuals’ perceptions of the childbirth process. According to a recent study conducted by Sultana et al.^6^, the utilization of cesarean birth in cases where there are medical grounds has demonstrated a significant reduction in both maternal and newborn mortality rates. According to the World Health Organization, it is recommended that the cesarean delivery rate falls within the range of 10–15%^7^. The global prevalence of cesarean deliveries has been on the rise, and Turkey stands out with one of the highest rates at 57.3%^8^. The aforementioned study conducted by Yazıcı Topçu and Aktaş^9^ demonstrates that this view also exerts an influence on maternal birth preferences. In order to encourage vaginal delivery and minimize the occurrence of elective cesarean sections, it is essential to assess the influence of traumatic birth perception on choices for birth methods^10^. Existing literature has investigated the correlation between traumatic birth perception and health literacy, prenatal attachment, and pregnancy stress in pregnant women^2,9,11^. However, there is currently a gap in research regarding the influence of traumatic birth perception on preferences for birth mode. The objective of this study is to examine the relationship between pregnant women's perceptions of traumatic birth and their preferences for the mode of delivery.

## METHODS

### Participants and study design

This study was designed as a cross-sectional, correlational investigation to examine the relationship between pregnant women's perceptions of traumatic birth and their preferences for the mode of delivery. The study was conducted in the Obstetrics and Gynecology Clinic of Osmaniye State Hospital, a 600-bed tertiary care hospital in Osmaniye, Turkey. Ethical approval for this study was obtained from the Osmaniye Korkut Ata University Scientific Research and Publication Ethics Committee on December 28, 2022 (Decision No: 2022/10/1). Institutional permission was also obtained from the hospital administration (E-77378720-799-211054512). Informed consent was obtained from all participants.

A preliminary investigation was undertaken to determine the appropriate sample size for the project. In accordance with the established guidelines for pilot studies as outlined in the literature, a cohort of 55 pregnant women who satisfied the specified inclusion criteria were successfully contacted^12^. The sample computation in this study utilized G-power 3.1.9.4, as indicated by the pilot study results. The present investigation utilized a 95% confidence level (1-α), a test power of 95% (1-β), and an effect size of f2=0.259 to calculate a minimum sample size of 77. In order to account for probable loss in the study, it was decided to increase the number of participants by 50%, resulting in a final sample size of 337 pregnant women.

### Location and characteristics of the study

The study population consisted of pregnant women who applied to the Obstetrics and Gynecology Clinic of Osmaniye State Hospital, a 600-bed tertiary care hospital in Osmaniye, Turkey, during the study period. The inclusion criteria were being 18 years or older, having a singleton pregnancy between the 20th and 40th gestational weeks, and having no high-risk pregnancy conditions. The exclusion criteria included pregnancy through assisted reproductive techniques and the presence of intellectual disabilities.

Participants who met the inclusion criteria were recruited through a non-probability random sampling method. Of the participants, 22% were primiparous and 78% were multiparous.

### Data collection tools

The Participant Information Form was developed by the researchers based on a comprehensive review of the literature and consisted of 14 questions. The form covered two main areas: sociodemographic characteristics and pregnancy and delivery-related factors. The sociodemographic section included questions regarding age, education level, employment status, spouse's education level and employment status, family type, place of residence, and income level. The pregnancy and delivery-related section assessed the number of pregnancies, parity, perceived social support, exposure to negative birth experiences shared by others, the preferred mode of delivery, and factors influencing this preference.

#### Traumatic Childbirth Perception Scale

The Traumatic Childbirth Perception Scale (TCPS), developed by Yalnız Dilcen et al., is a 13-item instrument designed to assess women's perception of traumatic birth experiences^13^. The scale is rated from 0 to 10 for each item, and the total score is calculated based on the mean score across all items. Higher scores on the scale indicate a greater perception of childbirth as traumatic. The scale's reliability was originally reported with a Cronbach's alpha coefficient of 0.90^13^. In this study, the internal consistency of the scale was found to be 0.86.

#### Belief Scale for Normal Delivery

The Belief Scale for Normal Delivery (BSND), developed by Ibici Akça and Aksoy Derya, consists of 24 items distributed across six subscales^10^. The total score ranges from 24 to 120. The scale measures pregnant women's beliefs and attitudes toward vaginal delivery, with higher scores indicating a stronger belief in and preference for vaginal birth. The original Cronbach's alpha coefficient was reported as 0.84^14^, while in the present study, it was calculated as 0.66.

### Data analysis

The data obtained from the study were analyzed using SPSS 25.0. Descriptive statistics, including frequency, percentage, mean, and standard deviation, were used to summarize numerical data. For comparisons, t-tests, Mann-Whitney U tests, one-way ANOVA, and multiple linear regression analysis were conducted.

### Data collection

Data were collected through face-to-face interviews conducted with pregnant women in a private setting. Before participation, the purpose of the study was explained in detail, and those who agreed to participate were included in the study.

## RESULTS

**Figure 1:**
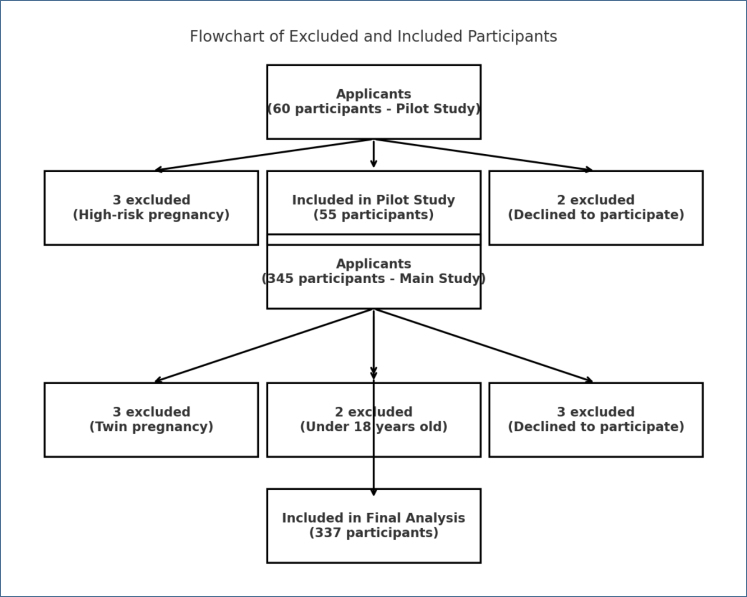
Flowchart of data collection and analysis process.

A flowchart was created to illustrate participants’ inclusion and exclusion process (Figure 1). Initially, 60 pregnant women were assessed for eligibility in the pilot study, with 5 excluded due to risk factors or refusal to participate. In the main study, 345 participants were screened and 8 were excluded based on predefined criteria. Ultimately, 337 participants were included in the final analysis.

The sociodemographic characteristics of the pregnant participants are summarized in Table 1. The majority of participants had an educational level below university, were unemployed, and had spouses with higher employment rates. Most participants lived in nuclear families, resided in urban areas, and reported their income as equal to their expenses (Table 1). Table 1 presents the differences in traumatic birth perception and beliefs about normal delivery based on employment status and residential area. Employed pregnant women had significantly higher traumatic birth perception scores than unemployed women. Additionally, normal birth belief scores were significantly higher among unemployed women and those living in districts (Table 1).

**Table 1:** Distribution of Traumatic Childbirth Perception Scale and Belief Scale for Normal Delivery by pregnant women's sociodemographic characteristics.

Sociodemographic characteristics		n (%)	TCPS		BSND	
			Median (IQR)	Significance	Mean (SD)	Significance
Education level						
	<University	249 (73.9)	80 (61–91)		72.5 (9.22)	
	≥University	88 (26.1)	79 (63–88)	p=0.371	71.9 (8.37)	p=0.862
Employment status						
	Employed	92 (27.3)	83.5 (77–91)		67.3 (7.81)	
	Unemployed	245 (72.7)	76 (55–90)	**p=0.001**	73.9 (8.75)	**p<0.001**
Spouse's education						
	<University	215 (63.8)	78 (56–90.5)		72.4 (9.32)	
	≥University	122 (36.2)	81 (70–90)	p=0.097	71.6 (8.4)	p=0.466
Spouse's employment						
	Employed	316 (93.8)	79.5 (61.5–90.5)		72.1 (8.91)	
	Unemployed	21 (6.2)	77 (56–89)	p=0.782	72.1 (10.42)	p=0.999
Family type						
	Nuclear family	301 (89.3)	80 (62–91)		72.1 (8.95)	
	Extended family	36 (10.7)	73.5 (54–81)	**p=0.005**	72.1 (9.46)	p=0.976
Residence						
	City	300 (89)	81 (64.5–91)		71.4 (8.88)	
	District	37 (11)	60 (43–65)	**p<0.001**	78.1 (7.63)	**p<0.001**
Income status						
	Income<expenses	66 (19.6)	77.5 (65–85)		70.7 (8.02)	
	Income=expenses	271 (80.4)	80 (60–91)	p=0.158	72.4 (9.2)	p=0.168

Mean Age=30.4±7.3, U=Mann-Whitney U test, t=independent t-test, TCPS: Traumatic Childbirth Perception Assessment Scale, BSND: Belief Scale for Normal Delivery; SD: standard deviation; IQR: interquartile range. Statistically significant values are indicated in bold.

A statistically significant difference was found between the perception of traumatic birth and social support during pregnancy. Women receiving social support had higher traumatic birth perception than those without support (p=0.001). Similarly, exposure to negative birth stories was linked to a higher perception of traumatic birth (p<0.001). Birth preference and influential factors in delivery decision-making were also associated with traumatic birth perception. Women preferring cesarean birth and those influenced by healthcare professionals had higher traumatic birth perception (p<0.001; p<0.001). A significant difference was observed between the number of pregnancies and belief in normal birth, with women having 1–3 pregnancies showing a stronger belief in normal birth than those with four or more (p=0.036). Furthermore, women inclined toward vaginal delivery and those influenced by their families had a stronger belief in normal birth than those favoring cesarean birth or influenced by healthcare professionals (p<0.001; p<0.001) (Table 2).

**Table 2:** Distribution of Traumatic Childbirth Perception Scale and Belief Scale for Normal Delivery by variables related to pregnancy and delivery mode.

Characteristics related to pregnancy and delivery mode		n (%)	TCPS		BSND	
			Median (IQR)	Test and significance	Mean (SD)	Test and significance
Pregnancy count						
	1–3	245 (72.7)	78 (60–90)	U=10,583.000	72.7 (8.82)	t=2.105
	4 and more	92 (27.3)	82 (63–91)	p=0.388	70.4 (9.27)	**p=0.036**
Receiving social support						
	Yes	287 (85.2)	80 (63–91)	U=4,970.500	72 (8.96)	t=-0.308
	No	50 (14.8)	71.5 (47–83)	**p=0.001**	72.5 (9.27)	p=0.758
Exposure to negative birth experience sharing in the environment						
	Yes	240 (71.2)	82 (63–92)	U=7,650.500	72 (9.08)	t=-0.234
	No	97 (28.8)	71 (52–81)	**p<0.001**	72.3 (8.81)	p=0.815
Delivery mode preference						
	Vaginal birth	97 (28.8)	55 (41–67)	U=4,389.000	81.5 (6.26)	t=16.283
	Cesarean section	240 (71.2)	83.5 (76–92)	**p<0.001**	68.3 (6.91)	**p<0.001**
Influential parameter in delivery mode preference						
	Physician	224 (66.5)	81.5 (63–92)	1>3, 2>3	71.7 (9)	3>1, 3>2
	Nurse/midwife	55 (16.3)	81 (76–87)	KW=26.057	69 (7.56)	F=11.009
	Family	58 (17.2)	63 (52–71)	**p<0.001**	76.5 (8.71)	**p<0.001**

U=Mann-Whitney U test, t=independent t-test, TCPS: Traumatic Childbirth Perception Assessment Scale, BSND: Belief Scale for Normal Delivery; SD: standard deviation. Statistically significant values are indicated in bold.

Table 3 presents a multiple linear regression analysis examining factors associated with pregnant women's belief in normal birth. The model was built using the enter method and included income status, exposure to negative birth experiences shared by other women, age, and the perception of traumatic birth as independent variables. Age was found to have a significant effect on belief in normal birth (p=0.001). A one-unit increase in age was associated with a 0.21-unit decrease in belief in normal birth scores.

**Table 3:** Relationship between scores on the normal birth belief scale and selected variables among pregnant women*.

BSND risk factors	BSND total scores						
	B (95%CI)	Beta	t	p	Zero-order	Partial
(Constant)	92.578 (87.332–97.824)		34.715	**<0.001**		
Income status (Reference: income than expenses)	1.178 (-1.003–3.358)	0,052	1,062	0.289	0.075	0.058
Sharing negative experiences about birth (Reference: yes)	−1.706 (−3.656–0.244)	−0.086	−1.721	0.086	0.013	−0,094
Age	−0.21 (−0.331 to −0.089)	−0.170	−3.415	**0.001**	−0.250	−0.184
TCPS	−0.196 (−0.243 to −0.149)	−0.419	−8.267	**<0.001**	−0.427	−0.413

* Multiple Linear Regression Analysis,

B: Unstandardized coefficient, Beta: Standardized coefficient, F=23.997, p=0.000, R=0.474, R^2^=0.224, AdjR^2^=0.215, SE=7.967, TCPS: Traumatic Childbirth Perception Assessment Scale, BSND: Belief Scale for Normal Delivery; CI: confidence interval. Statistically significant values are indicated in bold.

The overall model explained 21.5% of the variance in belief in normal birth (R^2^=0.224, Adjusted R^2^=0.215, p<0.001) (Table 3).

## DISCUSSION

This study investigated the factors influencing pregnant women's perception of traumatic birth and their belief in normal birth using the TCPS and BSND scales. The findings showed that social support and exposure to negative birth experiences significantly affected the traumatic birth perception. Multiple linear regression analysis revealed that income status, exposure to negative birth experiences, age, and traumatic birth perception collectively explained a significant proportion of the variance in belief in normal birth. The findings of this study indicate that pregnant women who are employed tend to consider the experience of childbirth as more traumatic. Altuntuğ et al.^15^ conducted a study with a sample size of 285 participants, of which 87.7% were nonworking individuals. The study findings revealed that 54.1% of the participants had diminished levels of traumatic birth perception. The findings of this study also revealed that employed women tended to see childbirth as a more distressing experience. In contrast, Yılmaz et al.^16^ discovered diminished levels of traumatic birth perception among employed individuals in a research endeavor that explored the correlation between personality qualities and traumatic birth perception. While the existing body of literature shows a range of outcomes, it is conceivable that an increase in educational attainment and improved accessibility to information among women in the workforce could potentially contribute to a heightened impression of trauma associated with their experiences.

Additionally, the findings of this study indicate that pregnant women who are employed exhibit a decreased inclination toward opting for vaginal birth in comparison to those who are not employed. In a study conducted by Begum et al.^17^ in Bangladesh, it was found that the majority of participants (98.3%) were not engaged in employment. The study also revealed that 65% of all births in the sample were delivered vaginally. The results of our study align with this conclusion since we observed that those who were not employed exhibited a stronger inclination for vaginal birth. Several studies conducted in different countries have shown that women choose vaginal delivery as a means to prevent a decrease in their socioeconomic status resulting from post-birth work limitations^18,19^. The different findings observed in these studies may be attributed to changes in the social support system, which can have an impact on both the decision-making process and the resulting outcomes.

Additionally, the findings of this study suggest that pregnant women who have a predisposition for cesarean birth experience elevated levels of traumatic birth perception. According to the findings of Demšar et al.^20^, pregnant women exhibiting elevated levels of birth anxiety demonstrated a greater propensity for opting for cesarean delivery. Furthermore, the findings of this study indicate a negative correlation between age and both belief in and propensity toward normal delivery. According to Loke et al.^21^, there was a higher propensity among women aged 36 and older to opt for elective cesarean delivery as opposed to vaginal birth. In contrast, the study conducted by Safari-Moradabadi et al.^22^ revealed a higher inclination among older women toward opting for vaginal birth. The multitude of these studies highlights the complex and varied nature of birth preference. Consistent with our main hypothesis, the findings of this study indicate that there is a negative correlation between pregnant women's perception of traumatic birth and their belief in and desire for regular birth. According to a study conducted by Weeks et al.^23^, there was a significant association between birthing anxiety and the preference for cesarean birth in Chile, with persons expressing childbirth anxiety being four times more likely to have a preference for cesarean birth. According to the findings of Hauck et al.^24^, those exhibiting elevated levels of birth anxiety displayed a preference for cesarean or epidural anesthesia-assisted normal birth. The findings of this investigation exhibit a resemblance to our research.

### Limitations

This study has several limitations that should be considered when interpreting the findings. First, the study was conducted in a single hospital setting, which may limit the generalizability of the results to the broader population of pregnant women. The findings may be influenced by the specific characteristics of the healthcare institution and the sociodemographic profile of the participants. Finally, the study did not account for potential confounding factors such as psychological history, previous birth experiences, or healthcare accessibility, which may have influenced the participants’ perceptions and beliefs. Future research should incorporate longitudinal designs and more diverse samples to strengthen the validity and applicability of these findings.

## CONCLUSION

This study highlights the significant factors influencing pregnant women's perception of traumatic birth and their belief in normal birth. The findings indicate that social support, exposure to negative birth experiences, age, and birth preference play a crucial role in shaping these perceptions. Women who received social support exhibited higher traumatic birth perception, while those exposed to negative birth experiences were more likely to have heightened traumatic birth perceptions. Additionally, age was found to be a determining factor in normal birth belief, with older women demonstrating lower scores. By addressing these influencing factors through enhanced healthcare provider education and support, maternity experiences can be improved. Future research should explore these dynamics further in diverse populations and longitudinal contexts to provide a deeper understanding of childbirth perceptions and preferences.

## Data Availability

The datasets generated and/or analyzed during the current study are available from the corresponding author upon reasonable request.
